# Altered gene expression and miRNA expression associated with cancerous IEC-6 cell transformed by MNNG

**DOI:** 10.1186/1756-9966-28-56

**Published:** 2009-04-28

**Authors:** Bo Zhang, Xukai Wang, Yan Wang

**Affiliations:** 1 Department of Medical Genetics, Third Military Medical University, Chongqing 400038, PR China; 2 Department of Cardiovascular, Daping hospital, Third Military Medical University, Chongqing 400042, PR China

## Abstract

**Background:**

Tumorigenesis is thought to be the consequence of gene mutation and disordered gene expression. However, the detailed molecular mechanism underlying the development and progress of colon cancer have not been elucidate completely. This study aimed to find out the genes associated with cancer biological pathways involved in transformation and tumorigenesis.

**Methods:**

Normal intestinal cell line 6 (IEC-6) cells were transformed to cancer cells by treatment with cancerogenic agent of N-methyl-N'-nitro-N-nitrosoguanidine (MNNG) and Phorbol 12-myristate 13 acetate (PMA). Then we investigated the altered gene expression of transformed IEC-6 cells by the microarray containing 113 genes associated with cancer pathway. Also the altered miRNAs of transformed IEC-6 cells were analyzed by array hybridization (miRCURY Array v9.2, Exiqon). The levels of acetylated histone H3 in transformed IEC-6 cells was evaluated by western blot.

**Results:**

Cell proliferation was significantly increased as IEC-6 cells were transformed and tumor xenografts could be detected in animals as transformed IEC-6 cells were inoculated subcutaneously in nude mice. Result of microarray showed nine genes were increased and two decreased, as well as 13 miRNA were increased and 97 decreased. Verification by real-time PCR implies that the data obtained from microarray analysis were reliable. Western blot showed the levels of acetylated histone H3 were increased dramatically after MNNG/PMA treatment.

**Conclusion:**

Our results showed many important biological pathways and miRNAs were involved in transformation and tumorigenesis of IEC-6 cells, which suggested the transformation of normal cells was involved with large mount of genetic and epigenetic variation.

## Background

Colon cancer is one of the most common cancers associated with considerable mortality and morbidity rates [1,2]. Most colorectal malignancies are sporadic, but a fraction of colon cancers occur in an inherited fashion. Familial adenomatous polyposis (FAP) is one of the best-characterized inherited colon cancers, with patients developing hundreds to thousands of preneoplastic colonic polyps in early adulthood [3]. Tumor suppressor APC was thus cloned as the causative gene for this disease. Other genes associated with colon cancer have already outlined, which causally interpret the development of inherited colon cancer syndrome [4]. As for sporadic cases, another series of genes account for the susceptibility of colon cancer.

Much effort was paid to address the cancer biological pathways such as cell apoptosis, cell cycle control and signal transduction in transformed cell models, in which carcinogens were applied [5]. Chemical carcinogens could be divided into two categories (initiators and promoters) based on the two-stage model of carcinogenesis, though criticism about this theory was still existed [6]. So the transformation of normal cells could be divided as two-stages of initiation and promotion [7]. Most initiators can be detected by various genotoxicity tests. However, the promoters, which do not damage DNA directly, can facilitate tumor development from initiated cells. Now, more and more chemicals have been identified as tumor promoters in experimental animals and in cell transformation models, and their molecular mechanisms have been undoubtedly elucidated [8]. Two of the most frequently used chemicals are MNNG and PMA. For example, BALB/c-3T3-cell was successfully transformed by MNNG and PMA treatment [9]. As a consequence result, transformed foci were the final outcome of transforming cells in a malignant state. This kind of transformation assay can detect both initiating and promoting activities, which might be a screening tool for detection of not only tumor initiators but also tumor promoters such as non-genotoxic carcinogens.

The process of adenoma growth and transformation was accompanied by cumulative mutations in genetic pathways that confer a growth advantage of colon cancer. These pathways included cell cycle controlling, cell signaling pathway, cell apoptosis and adhesion [10]. So the major challenge is to identify the molecular signatures that indicate increased likelihood for colon cancer progression. Most of importantly, it has been reported that microRNA (miRNAs) was involved in the development of caner [11,12]. Characteristic patterns of miRNAs expression are precisely regulated. Deviations from normal pattern of expression may play a role in diseases, such as in tumorigenesis and progress. Indeed, altered miRNAs expression has been reported in many types of cancer cells, although the functional significance of these changes has yet to be fully addressed [13,14]. As colon caner concerned, aberrantly expressed or mutation of individual miRNAs were reported [15-17]. For example, miR-143 and miR-145 consistently display reduced steady-state levels of the mature miRNAs at the adenomatous and cancer stages of colorectal neoplasia, by comparative analyzing of human samples. Furthermore, miR-143 and miR-145 would be potentially useful as diagnostic and therapeutic tools for colon cancer and other types of cancer [18,19]. With the accumulating evidences in the literature that new genes found to be implicated in colon cancer, the detailed molecular mechanism underlying the development and progress of colon cancer remains unknown.

To find out the genes associated with cancer biological pathways involved in transformation and tumorigenesis, we transformed normal IEC-6 cells to cancer cells by treatment with cancerogenic agent of MNNG and PMA. IEC-6 cell line was derived from normal rat intestinal epithelia [20]. We transformed IEC-6 cells, and identified the altered gene expression by rat Oligo GEArray microarray of the six biological pathways involved in transformation and tumorigenesis. At the same, we indentified the altered miRNAs of transformed IEC-6 cells by array hybridization. Our result gave clues to find out the molecular mechanism of development and progress of colon cancer.

## Materials and methods

### Cell culture, animal and reagents

Chemicals employed were obtained from the following sources: MNNG and PMA from Sigma Chemical Co. (St. Louis, MO, USA). These chemicals were dissolved in dimethyl sulfoxide (DMSO, from Sigma Chemical Co.) before addition to the cultures. The final concentration of DMSO was 0.1%. Antibodies against acetylated histone H3 and GAPDH were from SantaCruz (California, USA). The rat Oligo-GE-Array (9.2 version) was supplied Exiqon (Denmark).

Male Balb/c nude mice, 6–8 weeks of age, were obtained from The Animal Facility of Third Military Medical University (Chongqing, China). Animals were housed under controlled temperature, humidity and day-night cycle with food and water. All animal experiments were conducted according to the Cancer Statement for the Use of Animals in Cancer Research, and approved by the institutional committee for animal research of Third Military Medical University, Chongqing, China.

### Cell culture and cell transformation

IEC-6 cells (ATCC, USA) were cultured in DMEM (Logan, USA) containing 10% fetal calf serum (Hyclone), penicillin (100 U/mL), and streptomycin (100 μg/mL).

For cell transformation, exponentially growing cells were seeded at a density of 10^5 ^cells per 60-mm dish in 5 ml of culture medium. Twenty-four hours after seeding, the cells were treated with 1 μg/ml MNNG for 8 h and then grown in normal medium for 3 days. Then the cell culture was grown in a medium containing PMA at concentrations of 100 ng/ml for 3–4 days of promotion stage. The MNNG/PMA treatment was repeated 11 times and the finally treated IEC-6 cells were tested for transformation properties. Normal IEC-6 cells were used as negative control.

### Achorage dependence

The efficiency of colony formation in semisolid medium was measured by the procedure described by MacPherson [21]. Cells suspended in 3.0 ml of 0.3% agar with complete medium and were plated in 60-mm dishes over a layer of 0.7% agar containing complete medium. A final concentration was 1 × 10^4 ^cells per dish and allowed to harden. Plates were incubated at 37°C in a 5% CO_2 _humidifed atmosphere for 21 days and scored for clones. Colony formation efficiency in semisolid agar was expressed as the percentage of total cells that formed colonies containing at least 50 cells.

### Tumor development in nude mice

Normal or transformed IEC-6 cells were trypsinized and collected by centrifugation. Male Balb/c nude mice were inoculated subcutaneously with 5 × 10^5 ^IEC-6 cells in the dorsal aspect of the neck (4 mice in each group). Human colon cancer SW480 cells were used as positive control, and the same amount of cells were inoculated in nude mice as well. All the mice were further raised for 4–8 weeks, and the tumor weight was scored after the mice were kindly sacrificed. Dissected organs were kept in Bouin solution for 24 h, replaced with 70% ethanol for fixation, and subsequently immersed in paraffin, and l0 μm sections were cut with a microtome and stained with hematoxylin-eosin.

### Cell growth curve

Exponentially growing normal and transformed IEC-6 cells were cultivated in 96-well plate, with 1 × 10^4 ^cells in each well. Twelve hours later, ^3^H-TdR 7.4 × 10^4^Bq/ml was added into the culture media, and the plate was returned to the incubator for further cultivation. Cells were washed with cold PBS after discarding the culture media at indicated time. Excess ^3^H-TdR was removed by washing with 3 ml PBS. The cells were resuspended in 10% trichloroacetic acid (TCA) with vigorous vortexing. The cellular lysates were vacuum-filtered and then washed with cold 5% TCA. Incorporated ^3^H-TdR was measured in a liquid scintillation counter (Beckman LS5000TA, Fullerton, California, USA). The procedures were performed 3 times in duplicate 24-well culture dishes. Values are expressed as mean ± SEM.

### Gene expression studies using Rat Oligo GEArray

A rat Oligo GEArray microarray (Exiqon, Denmark) was employed to detect altered gene expression associated with cell transformation. RNA preparation: Total RNA was isolated from the cells of each group using TriPure reagent kit according to the manufacturer's protocol (Roche Diagnostics Co.). The integrity of RNA sample was assessed by viewing the ethidium bromide-stained 28 S and 18 S ribosomal RNA bands, and the purity of RNA sample was verified by the absorption ratio OD_260 nm_/OD_280 nm_. Equal amounts of RNA isolated from normal and transformed IEC-6 cells were pooled for the following microarray detections. 3 μg total RNA was reverse transcribed into Biotin-16-dUTP-labeled cDNA probes with the TrueLabeling-AMP method according to the manufacturer's instructions. The microarray membranes were pre-hybridized at 60°C for at least 2 h. Hybridization of the Biotin-labeled cDNA probes to the membranes was carried out at 60°C overnight with slow agitation in a hybridization oven. The hybridized membranes were washed in saline sodium citrate buffer. Then membranes were incubated with alkaline phosphatase-conjugated streptavidin, washed and incubated with the chemiluminescent substrate CDP-Star. Images of the membranes were acquired using the Chemidoc XRS system (Biorad Laboratories) and analyzed. The relative expression level of each gene was determined by comparing the signal intensity of each gene in the array after correction for background and normalization.

### microRNA chips

miRCURY LNA™ microRNA chips (Exiqon, Vedbaek, Denmark) were employed to detect altered miRNA expression associated with cell transformation. The chips (version 9.2) contained totally 2056 probes, including human, mouse and rat miRNA genes, in triplicate. Total RNA (2–4 μg) was 3'-end-labeled using T4 RNA ligase and a Cy3-labeled RNA linker by the following procedure: RNA in 2.0 μL of water was combined with 1.0 μL of CIP buffer and CIP (Cat#208021, Exiqon). The mixture was incubated for 30 min at 37°C, and was terminated by incubation for 3 min at 80°C. Then 3.0 μL of labeling buffer, 1.5 μL of fluorescent label, 2.0 μL of DMSO, 2.0 μL of labeling enzyme were added into the mixture. The labeling reaction was incubated for 1 h at 16°C, and terminated by incubation for 15 min at 65°C. The labeled RNA was then combined with hybridization buffer, herring sperm DNA and DEPC-treated water. The samples were first denatured for 1–2 min at 80°C and then hybridized to the microarray for 16–20 h at 65°C under a lifterslip. Post-hybridization washes were done using Wash bufer kit (Cat#208021, Exiqon), according to the instructions of the manufacturer.

### Real-time qPCR

RNA samples were extracted from normal and transformed IEC-6 cells. A total of 5 μg RNA was reverse transcribed to cDNA according to the manufacturer's directions (Roche Diagnostics, USA). Specific primers were designed using the Primer Express software (Applied Biosystems, USA) and were checked for gene specificity using NCBI/Blast (Table 1). In presence of SYBR I Green (BioFlux, Japanese) the primers were used to amplify the expressed cDNA of individual gene using the ABI 5700 real-time PCR system (Applied Biosystems, USA). The relative abundance of each gene was normalized by the expression level of the GAPDH, according to the formula: ΔΔCt = (Ct_sample_-Ct_ref_)_N_-(Ct_sample_-Ct_ref_)_T_, and the estimated expression ratio is equal to 2^ΔΔCt^. To quantify miRNA, total RNA was reverse transcripted using specific RT primers (Table 2), and subsequent PCR was performed as above. The relative abundance of each miRNA was normalized by the expression level of U6 RNA.

**Table 1:** Sequences of forward and reverse primers for real-time quantitative PCR

GeneBank no.	Gene		Sequence(5'-3')	Product (bp)
NM_001014786	**Ifna1**	Fwd	GTGACCTGCCTCATACTCATAACC	443
			
		Rev	GACTTCTGCTTTGACCACCTCCC	

NM_022197	**Fos**	Fwd	GAGAATCCGAAGGGAAAGGAATAA	252
			
		Rev	GTCAAGTCCAGGGAGGTCACAGA	

NM_012603	**Myc**	Fwd	TCCTGTACCTCGTCCGATTCCAC	495
			
		Rev	ACGCTTCAGCTCGTTTCTCCTCT	

NM_031334	**Cdh1**	Fwd	GCCATCGCCTACACCATCCTCAG	282
			
		Rev	ACGGGCACCGACCTCATTCTCAA	

NM_013135	**Rasa1**	Fwd	CTACAACACTTGCGAGTACCTTG	276
			
		Rev	GAACTGATTTCTGTAAACACCCATA	

**Table 2:** Specifice RT primer and PCR primers

Gene name	RT primer	PCR primers
U6	5':CGCTTCACGAATTTGCGTGTCAT	F:5'GCTTCGGCAGCACATATACTAAAAT R:5'CGCTTCACGAATTTGCGTGTCAT
rno-miR-22*	5':GTCGTATCCAGTGCGTGTCGTGGAGTCGGCAATTGCACTGGATACGACTAAAGCT	GSP: 5'GGGAGTTCTTCAGTGGCA R:5'CAGTGCGTGTCGTGGAGT
rno-miR-208	5':GTCGTATCCAGTGCGTGTCGTGGAGTCGGCAATTGCACTGGATACGACACAAGCT	GSP: 5'GGGGATAAGACGAGCAAAA R:5'CAGTGCGTGTCGTGGAGT

### Western Blot

A cell suspension of normal and transformed IEC-6 cells was centrifuged and the cell pellet was washed with ice-cold PBS. Total proteins were extracted with lysis buffer (150 mmol/L NaCl, 50 mmol/L Tris-HCl, pH 7.4, 2 mmol/L EDTA, 1% NP-40) containing protease inhibitors. Total protein (30 μg per lane) was resolved on SDS-polyacrylamide gel and transferred onto a nitrocellulose membrane and incubated with anti-acetylated histone H3 and anti-GAPDH antibodies (Santa Cruz, California), followed by incubation with corresponding secondary antibodies. The bands were visualized by using the enhanced chemiluminescence system (Pierce, Rockford, IL). To validate the reproducibility, the tests were repeated for at least 3 times.

### Statistical analysis

Statistical analysis was performed using the independent 2-tailed t-test. All *P *values were two-tailed and considered statistically significantly if less than 0.05. Means, standard errors, and P values were calculated using SPSS version 11.0 for Windows.

## Results

### Cell transformation of IEC-6 cells

The method has been well established for cell transformation of normal cells with MNNG and PMA. We treated IEC-6 cells with MNNG and PMA for 12 times. After the final treatment, we detected the colony formation in semisolidified agarose of normal and MNNG/PMA treated IEC-6 cells. Transformed foci of normal IEC-6 cells were 0.02% and that of MNNG/PMA treated IEC-6 cells were 0.37%. MNNG/PMA treatment markedly enhanced the production of transformed foci (Table 3; *p *< 0.01).

**Table 3:** Transformation of IEC-6 cells by MNNG and PMA^1^.

Cell type	dishes	Number of clonies	Clong efficiency in soft agar(%)
normal	4	2 ± 0.1	0.02
MNNG/PMA	4	37 ± 0.2	0.37*

*p < 0.01 compared to untreated cells.

**Figure 1:**
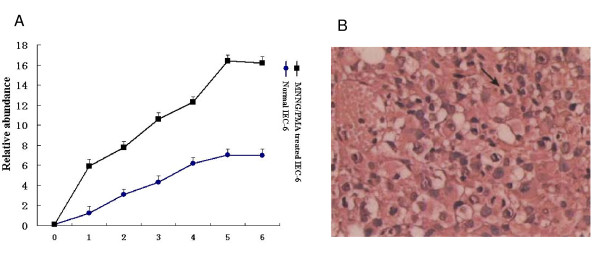
**Transformation of normal ICE-6 cells**. (A) Cell growth curves of normal and MNNG/PMA treated IEC-6 cells. (B) Histologically analysis of tumor xenografts inoculated with transformated IEC-6 cells.

Then we detected the cell growth curve of normal and MNNG/PMA treated IEC-6 cells. Cell proliferation was determined by ^3^H-TdR, which indicated the DNA synthesis. As shown in Fig. 1, cell growth of MNNG/PMA treated IEC-6 cells was significantly increased, compared with that of normal IEC-6 cells. The increased cell growth was coincident with the property of cancer cells. To further confirm its cancerous character, MNNG/PMA treated IEC-6 cells were inoculated subcutaneously in nude mice. As expected, tumor xenografts were detected in all animals 4 weeks later, which was coincident with the result of human cancer cell SW480. However, no tumor xenograft was visible in mice inoculated with normal IEC-6 cells even 8 weeks after inoculation. Fig. 1b showed the tumors were low- differentiated carcinomas. Histologically, the tumor cells of xenografts were arranged in flakiness and nest with round or polygon in shape. Tumor giant cells and mitotic phases could be seen. This suggested MNNG/PMA treated IEC-6 cells had been fully transformed.

### Changes of gene expression detected by microarray analysis

To elucidate the molecular mechanisms involved in cell transformation of IEC-6 cells, the rat Oligo GEArray microarray was used to identify genes with altered expression level after cell tranformation, compared with its normal controls. The microarray comprised 113 genes representative of the six biological pathways involved in transformation and tumorigenesis. As determined by repetitive microarray evaluations, a total of 11 genes exhibited differential expression of more than 2.0-fold compared with normal IEC-6 cells. Of these, nine genes were up-regulated and 2 were down-regulated (Table 4). The category of altered genes included apoptosis and cell senescence (Cflar, Bax), cell cycle control and DNA damage repair (Mdm2, Ccne1), angiogenesis (Ifna1, Egfr), adhesion (Itgav, Cdh1) and signal transduction (Fos, Myc, Rasa1).

**Table 4:** Differentially expressed genes related to cell transformation

GeneBank no	Symbol	Description	Ratio
NM_057138	Cflar	CASP8 and FADD-like apoptosis regulator,	2.06
NM_017059	Bax	Bcl2-associated X protein,	2.23
XM_235169	Mdm2	Transformed mouse 3T3 cell double minute 2,	2.73
XM_574426	Ccne1	Cyclin E	2.17
NM_001014786	**Ifna1**	Interferon-alpha 1	7.38
NM_031507	Egfr	Epidermal growth factor receptor	2.50
NM_022197	Fos	FBJ murine osteosarcoma viral oncogene homolog	6.50
NM_012603	**Myc**	Myelocytomatosis viral oncogene homolog (avian)	3.43
XM_230950	Itgav_predicted	Integrin alpha V (predicted)	3.22
NM_031334	**Cdh1**	Cadherin 1	0.07
NM_013135	**Rasa1**	RAS p21 protein activator 1	0.37

### Verification of differential expression genes by real-time PCR

**Figure 2:**
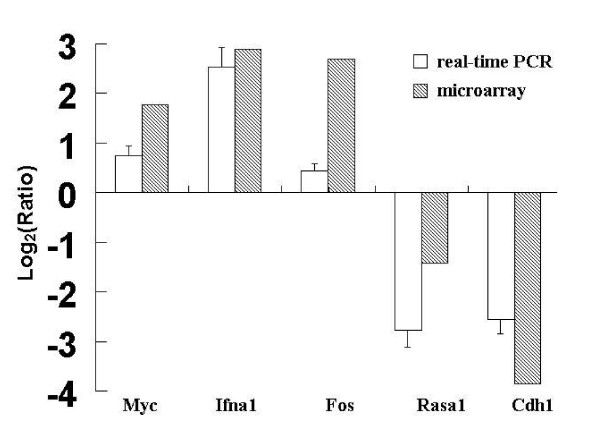
**Comparison of data obtained by real-time PCR and microarray analysis in transformed and normal IEC-6 cells**. Using the housekeeping GPADH gene as a reference gene, five selected genes were assessed for expression at the mRNA level by Real-time PCR. The ratio, representing the relative value of the gene expression level, was expressed as a logarithm (log_2_). Corresponding values obtained by microarray analysis were presented for comparison.

To confirm and validate the results obtained from microarray, we analyzed the expression of selected differentially expressed genes by real-time qPCR. Six genes were selected from the up-regulated and downregulated genes because of its ratio and putative gene functions. The ratios representing gene expression changes were log_2_-transformed in the histograms for these genes. The validation experiments showed expression patterns of other genes comparable to the microarray data (Fig. 2). This implies that the data obtained from microarray analysis were reliable.

### Changes of miRNAs expression

To determine the alteration of miRNA expression in transformed IEC-6 cells, total RNA samples from normal and transformed IEC-6 cells were isolated and hybridized to miRNA microarrays, comprising LNA-modified probes for all rat miRNAs in release 9.2 of the miRBase microRNA Registry. Expression profiling showed that a large set of miRNAs was expressed in IEC-6 cells. In agreement with other reports, several miRNAs, including miR-320, miR-494, miR-503, and members of the let-7 family, were highly expressed in IEC-6 cells, giving strong hybridization signals on the miRNA arrays. The top 5 miRNAs, which were highly expressed in IEC-6 cells, were miR-320, miR-494, miR-503, miR-185 and miR-206. Among them, the expression of miR-185 was altered in transformed IEC-6 cells. Totally, 20 miRNAs showed evidence of being differentially expressed within the transformed IEC-6 suggesting that they might be associated with the process of cell tranformation. For example, miR-208, miR-9, let-7a, 7b, and miR-22* were found to be up-regulated in transformed IEC-6 cells, whereas miR-539, miR-181d, and miR-146a were down-regulated. Additionally, the expressions of five miRPlus were also altered in transformed IEC-6 cells, although their identities have not been fully confirmed. The results on miRNAs displaying more or less than twofold in transformed IEC-6 cells compared to its normal controls were summarized in Table 5.

**Table 5:** Fold change in microRNAs in IEC-6 cells after transformation.

miRNA	Localization	Normal	Transformed	Ratio
miRPlus_17843	ND^a^	103.8	45.7	0.44
miRPlus_17858	ND^a^	109.5	41.5	0.38
miRPlus_17896	ND^a^	10457.5	27921.5	2.67
miRPlus_30317	ND^a^	137.5	782.4	5.69
miRPlus_30908	ND^a^	8473.3	19149.7	2.26
rno-let-7a	Intergenic, 17p14	10423.0	24709.6	2.37
rno-let-7b	Intergenic, 7q34	13462.8	42003.9	3.12
rno-miR-208	Intron, 15p13	11755.5	38910.7	3.31
rno-miR-9	Intergenic, 2q34	10761.0	28839.5	2.68
rno-miR-22*	Intergenic, 10q24	3401.3	8333.2	2.45
rno-miR-194	Intron, 13q26	1083.5	2405.4	2.22
rno-miR-126	Intron, 3p13	2880.7	6049.5	2.10
rno-miR-185	Intron, 11q23	34540.0	70461.6	2.04
rno-miR-217	Intergenic, 14q22	359.7	748.2	2.08
rno-miR-184	Intron, 8q31	23366.7	48656.7	2.08
rno-miR-146a	Intergenic, 10q21	130.5	57.4	0.44
rno-miR-292-5p	Intergenic, 1q12	107.5	41.9	0.39
rno-miR-30e	Intron, 5q36	115.8	55.6	0.48
rno-miR-539	Intergenic, 6q32	141.8	68.1	0.48
rno-miR-181d	Intergenic, 19q11	489.3	225.1	0.46

a: not determined.

**Figure 3:**
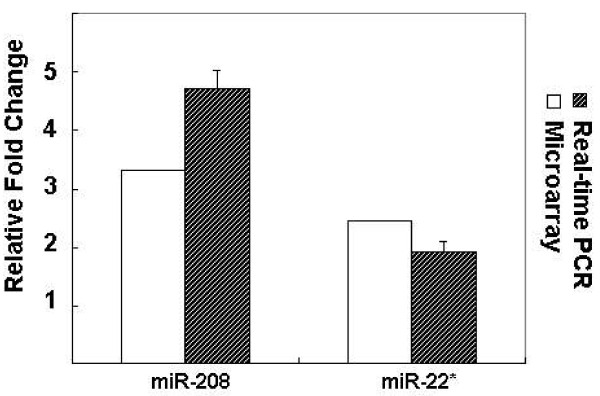
**Comparison of data obtained by real-time PCR and microarray analysis in transformed and normal IEC-6 cells**. Using the U6 gene as a reference gene, 2 selected miRNA genes were assessed for expression by Real-time PCR. Corresponding values obtained by microarray analysis were presented for comparison.

To validate the data of microarray, we partially assessed the expression of two miRNAs gene by real-time RT-PCR, using the same RNA samples that were applied to the microarrays. We were interested in those miRNAs, which gave strong hybridization signals, and were up-regulated in transformed cells. So the expression of miR208 and miR22* was chosen to be validated. As shown in Fig. 3, we found strong correlation between microarray profiling and real-time RT-PCR data. This implied that the data obtained from microarray analysis were partially reliable at least.

### Changes of acetylation status of histone H3

**Figure 4:**
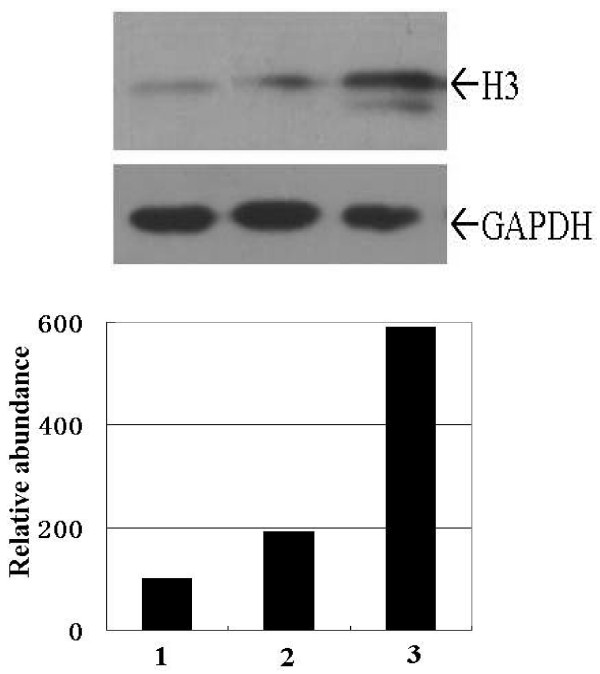
**Western blot analisis of acetylated histone H3 in IEC-6 cells**. Total protein (30 μg per lane) was resolved on SDS-polyacrylamide gel and transferred onto a nitrocellulose membrane and incubated with anti-acetylated histone H3 and anti-GAPDH antibodies. The bands were visualized by using the enhanced chemiluminescence system. The relative abundances of acetylated histone H3 was normalized by that of GAPDH. Lane1: normal IEC-6 cells; Lane2: IEC-6 cells treated with MNNG/PMA for 6 times; Lane2: IEC-6 cells treated with MNNG/PMA for 12 times.

It has been reported that aberrant acetylation of histone was involved in transformation and tumorigenesis. As large mount of genes and miRNAs were differential expressed in transformed IEC-6 cells, we wondered whether acetylation status of histone was also changed. Total proteins of normal and MNNG/PMA treated IEC-6 cells were isolated and detected by western blot with specific antibody against acetylated histone H3. Result showed the levels of acetylated histone H3 were low in normal IEC-6 cells, and it increased dramatically after MNNG/PMA treatment (Fig. 4). Furthermore, more times treated with MNNG/PMA, more increase of acetylated histone H3 was observed. This indicated that MNNG/PMA treatment leaded to increased level of acetylated histone H3, and thus altered gene expression.

## Discussions

Substantial advances in our understanding during the past decades have led to a complete understanding of the role of both environmental and genetic factors in colorectal cancer pathogenesis. It has been well demonstrated that the its development and progress are associated with deregulation of many genes, as well as mutational activation of oncogenes and loss of function of tumor suppressor genes [22]. And its tumorigenesis is also a process of multistage of hit. As a multiple factor disease, complex genetic pathways are involved in colorectal cancer. The most troublesome bewilderment is that different profile of mutant genes leads to the same clinical phenotype. So the detailed molecular mechanism of colorectal cancer has not been fully understood.

Many important biological processes were involved in transformation and tumorigenesis, including cell cycle control, DNA damage repair, cell apoptosis and signal transduction. The rat Oligo GEArray microarray profiles the expression of 113 genes representative of the six biological pathways involved in transformation and tumorigenesis. It has been applied in many experiments [23,24]. Our results showed that most of the six biological pathways were involved in transformation of IEC-6 cells. This indicated that transformation were the consequence of multiple deregulations of genes. Among the genes differentially expressed, some were also found altered in tumor by other researchers. Our experiment showed that c-fos was up-regulated greatly in transformed cells. Many experimental and clinical data indicated that c-fos expression plays a role in the progression of several types of carcinomas [25]. Increased expression of c-fos was also found to be associated with metastasising ability of metastatic colorectal cancer by cDNA macroarray analysis [26]. Another gene increased greatly in transformed IEC-6 cells was Ifna1, which played an important role in angiogenesis of tumor. Ifna1 plays a pivotal role not only in antiviral immunity but also in the surveillance of cancer development. It has showed that Ifna1 was endowed with marked antiangiogenic activity in breast cancer and ovarian cancer models, and considered as a suitable candidate gene for this therapeutic approach [27,28]. However, the functional significance of increased Ifna1 in transformed IEC-6 cells was unclear. Cdh1 was showed to be down regulated in transformed IEC-6 cells, which was coincident with others' findings. Cdh1 played a key role in cell-cell adhesion. Inactivation of the cdh1 mediated cell adhesion system was a common finding in human cancers, indicating that cdh1 function as tumor suppressor and invasion suppressor genes [29,30].

miRCURY microRNA chips contained totally 2056 probes, including human, mouse and rat miRNA genes. So it has been broadly applied in many research works [31,32]. Our data indicated several miRNAs were highly expressed in IEC-6 cells and 20 miRNAs showed evidence of being differentially expressed within the transformed IEC-6 cells. Among these differentially expressed miRNAs, we verified the alteration of miR-208 and miR-22*. miR-208 is encoded by intron 27 of the human and mouse MHC gene. Consistent with the specific expression of MHC in the heart and the pulmonary myocardium, miR-208 is expressed specifically in the heart and at trace levels in the lung [33]. The relationship between miR-208 and tumorigenesis was not clear and needed further study. miR-22* and miR-22 are the alternative mature type of their primary precursors. Increased miR-22 was found in erythropoiesis, and it was predicted to target genes involved in cell development and differentiation [34]. Our result showed miR-22* was increased, but not miR-22. This suggested that the maturation of primary precursor was selectively processed. Partial differential expressed miRNAs in transformed IEC-6 cells were consistent with the results of others. *Gottardo F et al *found significant up-regulation of miR-185 in renal cell carcinoma compared to normal kidney [35]. Many targets have been reported for miR-185, including genes of the proto-cadherin gene cluster. However, we didn't find the directly relationship between altered miRNAs and the specific genes in our experiment.

Our results suggested that transformation of IEC-6 cells did not derived from a single gene, but rather through accumulated changes in the expression of several different genes involved in many biological pathways. So it was necessary to find out the reason why genes were deregulated at chromosomal levels. In the past few decades, evidence has accumulated showing that modifications of histone acetylation status have a central role in carcinogenesis [36-38]. Aberrant activation of histone deacetylases in tumour cells leads to transcriptional deregulation of a diverse set of genes mainly involved in the regulation of proliferation, migration, angiogenesis, and invasion. In this study, we showed that the increased level of acetylation of histone H3 was observed in transformed IEC-6 cells. We speculated that treatment of MNNG/PMA could modify the histone acetylation status, and gave rise to altered gene expression of transformed cells.

## Conclusion

This study showed IEC-6 cells were successfully transformed and the corresponding altered gene expression was compared by microarray analysis. This strategy provided an efficient resolution to analyze the molecular mechanism of transformation and tumorigenesis of colon cancer. The preliminarily verified genes will of course be further studied in order to determine its functions in tumorigenesis of cancers. Our results showed many important biological pathways and miRNAs were involved in transformation and tumorigenesis of IEC-6 cells. This suggested the transformation of normal cell was involved with large mount of genetic and epigenetic variation.

## Competing interests

The authors declare that they have no competing interests.

## Authors' contributions

BZ participated in designing the study, western blot analysis, real-time qPCR and data analysis of microarray. XW participated in the design of the study and conducted cell line transformation and cell experiments. YW conceived of the study, participated in its design and coordination, and drafted the manuscript. All authors read and approved the final manuscript.

## References

[B1] Rougier P, Andre T, Panis Y, Colin P, Stremsdoerfer N, Laurent-Puig P (2006). Colon cancer. Gastroenterol Clin Biol.

[B2] Boursi B, Arber N (2007). Current and future clinical strategies in colon cancer prevention and the emerging role of chemoprevention. Curr Pharm Des.

[B3] Kinzler KW, Vogelstein B (1996). Life (and death) in a malignant tumour. Nature.

[B4] Kaz AM, Brentnall TA (2006). Genetic testing for colon cancer. Nat Clin Pract Gastroenterol Hepatol.

[B5] Asada S, Sasaki K, Tanaka N, Takeda K, Hayashi M, Umeda M (2005). Detection of initiating as well as promoting activity of chemicals by a novel cell transformation assay using v-Ha-ras-transfected BALB/c 3T3 cells (Bhas 42 cells). Mutat Res.

[B6] Iversen OH (1995). Of mice and men: a critical reappraisal of the two-stage theory of carcinogenesis. Crit Rev Oncog.

[B7] Breheny D, Zhang H, Massey ED (2005). Application of a two-stage Syrian hamster embryo cell transformation assay to cigarette smoke particulate matter. Mutat Res.

[B8] Ao L, Liu JY, Gao LH, Liu SX, Yang MS, Huang MH, Cao J (2008). Differential expression of genes associated with cell proliferation and apoptosis induced by okadaic acid during the transformation process of BALB/c 3T3 cells. Toxicol In Vitro.

[B9] Tsuchiya T, Umeda M (1997). Relationship between exposure to TPA and appearance of transformed cells in MNNG-initiated transformation of BALB/c 3T3 cells. Int J Cancer.

[B10] Sabates-Bellver J, Flier LG Van der, de Palo M, Cattaneo E, Maake C, Rehrauer H, Laczko E, Kurowski MA, Bujnicki JM, Menigatti M, Luz J, Ranalli TV, Gomes V, Pastorelli A, Faggiani R, Anti M, Jiricny J, Clevers H, Marra G (2007). Transcriptome profile of human colorectal adenomas. Mol Cancer Res.

[B11] Zhang B, Pan X, Cobb GP, Anderson TA (2007). microRNAs as oncogenes and tumor suppressors. Dev Biol.

[B12] Skaftnesmo KO, Prestegarden L, Micklem DR, Lorens JB (2007). MicroRNAs in tumorigenesis. Curr Pharm Biotechnol.

[B13] Calin GA, Liu CG, Sevignani C, Ferracin M, Felli N, Dumitru CD, Shimizu M, Cimmino A, Zupo S, Dono M, Dell'Aquila ML, Alder H, Rassenti L, Kipps TJ, Bullrich F, Negrini M, Croce CM (2004). MicroRNA profiling reveals distinct signatures in B cell chronic lymphocytic leukemias. Proc Natl Acad Sci USA.

[B14] Calin GA, Dumitru CD, Shimizu M, Bichi R, Zupo S, Noch E, Aldler H, Rattan S, Keating M, Rai K, Rassenti L, Kipps T, Negrini M, Bullrich F, Croce CM (2002). Frequent deletions and down-regulation of micro-RNA genes miR15 and miR16 at 13q14 in chronic lymphocytic leukemia. Proc Natl Acad Sci USA.

[B15] Nakajima G, Hayashi K, Xi Y, Kudo K, Uchida K, Takasaki K, Yamamoto M, Ju J (2006). Non-coding MicroRNAs hsa-let-7g and hsa-miR-181b are Associated with Chemoresponse to S-1 in Colon Cancer. Cancer Genomics Proteomics.

[B16] Lanza G, Ferracin M, Gafà R, Veronese A, Spizzo R, Pichiorri F, Liu CG, Calin GA, Croce CM, Negrini M (2007). mRNA/microRNA gene expression profile in microsatellite unstable colorectal cancer. Mol Cancer.

[B17] Akao Y, Nakagawa Y, Naoe T (2006). let-7 microRNA functions as a potential growth suppressor in human colon cancer cells. Biol Pharm Bull.

[B18] Akao Y, Nakagawa Y, Naoe T (2007). MicroRNA-143 and -145 in colon cancer. DNA Cell Biol.

[B19] Akao Y, Nakagawa Y, Naoe T (2006). MicroRNAs 143 and 145 are possible common onco-microRNAs in human cancers. Oncol Rep.

[B20] Ran XZ, Su YP, Wei YJ, Ai GP, Cheng TM, Lin Y (2001). Influencing factors of rat small intestinal epithelial cell cultivation and effects of radiation on cell proliferation. World J Gastroenterol.

[B21] MacPherson I, Montagnier I (1964). Agar suspension culture for the selective assay of cells transformed by polyoma virus. Virology.

[B22] Early DS, Fontana L, Davidson NO (2008). Translational approaches to addressing complex genetic pathways in colorectal cancer. Transl Res.

[B23] Mangan SH, Campenhout AV, Rush C, Golledge J (2007). Osteoprotegerin upregulates endothelial cell adhesion molecule response to tumor necrosis factor-alpha associated with induction of angiopoietin-2. Cardiovasc Res.

[B24] Zhu R, Xu R, Jiang X, Cai Y, Zou Y, Du M, Qin L (2007). Expression profile of cancer-related genes in human adult bone marrow-derived neural stemlike cells highlights the need for tumorigenicity study. J Neurosci Res.

[B25] Milde-Langosch K (2005). The Fos family of transcription factors and their role in tumourigenesis. Eur J Cancer.

[B26] Saito N, Kameoka S, Furukawa R (2007). Gene profile analysis of colorectal cancer cell lines by cDNA macroarray. Oncol Rep.

[B27] Indraccolo S, Moserle L, Tisato V, Gola E, Minuzzo S, Roni V, Persano L, Chieco-Bianchi L, Amadori A (2006). Gene therapy of ovarian cancer with IFN-alpha- producing fibroblasts: comparison of constitutive and inducible vectors. Gene Ther.

[B28] De Boüard S, Guillamo JS, Christov C, Lefévre N, Brugières P, Gola E, Devanz P, Indraccolo S, Peschanski M (2003). Antiangiogenic therapy against experimental glioblastoma using genetically engineered cells producing interferon-alpha, angiostatin, or endostatin. Hum Gene Ther.

[B29] Qian ZR, Sano T, Yoshimoto K, Asa SL, Yamada S, Mizusawa N, Kudo E (2007). Tumor-specific downregulation and methylation of the CDH13 (H-cadherin) and CDH1 (E-cadherin) genes correlate with aggressiveness of human pituitary adenomas. Mod Pathol.

[B30] Nikuseva-Martic T, Beros V, Pecina-Slaus N, Pecina HI, Bulic-Jakus F (2007). Genetic changes of CDH1, APC, and CTNNB1 found in human brain tumors. Pathol Res Pract.

[B31] Castoldi M, Schmidt S, Benes V, Noerholm M, Kulozik AE, Hentze MW, Muckenthaler MU (2006). A sensitive array for microRNA expression profiling (miChip) based on locked nucleic acids (LNA). RNA.

[B32] Castoldi M, Schmidt S, Benes V, Hentze MW, Muckenthaler MU (2008). miChip: an array-based method for microRNA expression profiling using locked nucleic acid capture probes. Nat Protoc.

[B33] van Rooij E, Sutherland LB, Qi X, Richardson JA, Hill J, Olson EN (2007). Control of stress-dependent cardiac growth and gene expression by a microRNA. Science.

[B34] Choong ML, Yang HH, McNiece I (2007). MicroRNA expression profiling during human cord blood-derived CD34 cell erythropoiesis. Exp Hematol.

[B35] Gottardo F, Liu CG, Ferracin M, Calin GA, Fassan M, Bassi P, Sevignani C, Byrne D, Negrini M, Pagano F, Gomella LG, Croce CM, Baffa R (2007). Micro-RNA profiling in kidney and bladder cancers. Urol Oncol.

[B36] Shukla V, Vaissière T, Herceg Z (2008). Histone acetylation and chromatin signature in stem cell identity and cancer. Mutat Res.

[B37] Allen A (2007). Epigenetic alterations and cancer: new targets for therapy. IDrugs.

[B38] Sigalotti L, Fratta E, Coral S, Cortini E, Covre A, Nicolay HJ, Anzalone L, Pezzani L, Di Giacomo AM, Fonsatti E, Colizzi F, Altomonte M, Calabrò L, Maio M (2007). Epigenetic drugs as pleiotropic agents in cancer treatment: biomolecular aspects and clinical applications. J Cell Physiol.

